# Carrier frequency and incidence estimation of *RPE65*-associated inherited retinal diseases in East Asian population by population database-based analysis

**DOI:** 10.1186/s13023-022-02566-5

**Published:** 2022-11-09

**Authors:** Eun Hye Cho, Jong Eun Park, Taeheon Lee, Kyeongsu Ha, Chang-Seok Ki

**Affiliations:** 1 Department of Laboratory Medicine, Kangbuk Samsung Hospital, Sungkyunkwan University School of Medicine, Seoul, Republic of Korea; 2 Department of Laboratory Medicine, Hanyang University Guri Hospital, Hanyang University College of Medicine, Hanyang University Guri Hospital, 153, Gyeongchun-ro, 11923 Guri-si, Gyeonggi-do Republic of Korea; 3 GC Genome, Yongin, Republic of Korea

**Keywords:** *RPE65*-associated IRD, *RPE65*, gnomAD, KRGDB, Carrier frequency, East Asian

## Abstract

**Background:**

Inherited retinal diseases (IRDs) are clinically and genetically heterogenous disorders leading to visual impairment and blindness. Because gene therapy for *RPE65*-associated IRDs was recently approved, it is necessary to predict the carrier frequency and prevalence for *RPE65*-associated IRDs. This study aimed to analyze the carrier frequency and expected incidence of *RPE65*-associated IRDs in East Asians and Koreans using exome data from the Genome Aggregation Database (gnomAD) and the Korean Reference Genome Database (KRGDB).

**Methods:**

We analyzed 9,197 exomes for East Asian populations from gnomAD comprising 1,909 Korean and 1,722 Korean genomes from KRGDB. All identified *RPE65* variants were classified according to the 2015 American College of Medical Genetics and Genomics and the Association for Molecular Pathology guidelines.

**Results:**

The total carrier frequencies of East Asians and Koreans from both gnomAD and KRGDB were 0.10% (11/10,919) and 0.06% (2/3,631), respectively. The estimated incidence of *RPE65*-associated IRDs was 1/3,941,308 in East Asians and 1/13,184,161 in Koreans.

**Conclusion:**

This study identified carrier frequencies of *RPE65*-associated IRDs in East Asians and Koreans using gnomAD and KRGDB. We confirmed that the carrier frequency of *RPE65*-associated IRDs patients was low in Koreans among all East Asian populations, and the incidence was also predicted to be lower than in other East Asian populations. The variant spectrum of *RPE65* gene in East Asian and Korean populations differed greatly from those of other ethnic groups.

## Background

Inherited retinal diseases (IRDs) are clinically and genetically heterogenous disorders leading to visual impairment and blindness and affect approximately one in 2,000 individuals worldwide [1]. IRDs include several phenotypes, of which retinitis pigmentosa (RP) is the most common form and Leber congenital amaurosis (LCA) is the most severe form. To date, 280 genes have been discovered that are associated with IRDs [2].

*RPE65* encodes retinoid isomerohydrolase, which is responsible for converting all-*trans*-retinyl ester to 11-*cis*-retinol [3]. *RPE65* is associated with three IRD phenotypes, autosomal recessive LCA 2 (MIM 204,100), autosomal recessive RP 20 (MIM 613,794), and autosomal dominant RP 87 with choroidal involvement (MIM 618,697) and accounts for 2–16% of LCA and 1.02–2.7% of autosomal recessive RP [4].

On December 19, 2017, Voretigene neparvovec-rzyl (VN, LUXTURNA, Spark Therapeutics, Philadelphia, PA, USA) was approved by the Food and Drug Administration (FDA) for the treatment of *RPE65*-associated IRDs, becoming the first FDA-approved gene therapy for a genetic disease [5]. In Korea, it was also approved by the Ministry of Food and Drug Safety of Korea in September 2021. Therefore, it is important to know the carrier frequency of *RPE65* to predict the incidence of *RPE65*-associated IRDs, which is useful information for evaluating the potential number of patients for VN therapy.

The Genome Aggregation Database (gnomAD) is a widely used genomic database worldwide, and gnomAD is consists of 125,748 exomes and 4,359 genomes [6]. The gnomAD contains exome data collected from 9,197 East Asians, including 1,909 Koreans. It is suitable for East Asian studies as it has the largest amount of data from East Asians among the public genomic databases. The Korean Reference Genome Database (KRGDB) is a large-scale variant database of whole genome sequencing data of 1,722 Koreans and contains single nucleotide and short insertion/deletion variants [7]. The *RPE65* gene variant was interpreted according to the 2015 American College of Medical Genetics and Genomics and Association for Molecular Pathology guidelines (2015 ACMG/AMP guidelines), which has been widely adopted in clinical practice [8]. In this study, we analyze the carrier frequency of *RPE65* and expected incidence of *RPE65*-associated IRDs in East Asian populations using the population database from the gnomAD and KRGDB through 2015 ACMG/AMP guidelines.

## Methods

### East asian population data

Three representative population databases were used to analyze the carrier frequency of the *RPE65* gene in East Asians. The gnomAD data (v2.1.1) for the *RPE65* gene was obtained from gnomAD (https://gnomad.broadinstitute.org/, accessed on 13 September 2021). We analyzed 9,197 East Asian exomes of which 1,909 were from Koreans, 76 were from Japanese, and 7,212 were from other East Asian populations. The filtered variants that were flagged in gnomAD as failing ‘InbreedingCoeff,’ ‘AC0,’ or ‘RF’ QC filters were excluded from the analysis. The KRGDB was used as the Korean database, and the database consists of 1,722 Koreans (http://coda.nih.go.kr/coda/KRGDB/index.jsp, accessed on 25 September 2021).

### Variant classification and statistical analysis

All variants were interpreted using the 2015 ACMG/AMP guidelines and Sequence Variant Interpretation (SVI) general recommendations for ACMG/AMP criteria by ClinGen (https://clinicalgenome.org/working-groups/sequence-variant-interpretation/, accessed on 1 November 2021). The 2015 ACMG/AMP guidelines recommend the classification of variants into five categories: pathogenic variants (PV), likely pathogenic variants (LPV), variants of uncertain significance, likely benign variants, and benign variants. REVEL [9] and SpliceAI [10] were used to predict variant pathogenicity. All variants identified in gnomAD were additionally classified according to the Human Gene Mutation Database (HGMD) and ClinVar. The HGMD professional database (http://www.hgmd.org/, release 2021.04) is a comprehensive collection of germline variants categorized into six categories. ClinVar (https://www.ncbi.nlm.nih.gov/clinvar/, accessed on 5 February 2022) is a freely available archive that provides the classification of variants interpreted by clinical laboratories.

### *RPE65* carrier frequency and incidence estimation

East Asian and Korean carrier frequencies were calculated for *RPE65* gene using gnomAD and KRGDB. We used those classified as PV and LPV according to the 2015 ACMG/AMP guidelines, the disease-causing variant (DM) in HGMD and those classified as PV and LPV in ClinVar for carrier frequency analysis. Thereafter, we estimated the incidence of *RPE65*-associated IRDs based on frequency and the Hardy–Weinberg equilibrium principle (1 = *p*^2^ + 2*pq* + *q*^2^). The Hardy-Weinberg equilibrium states that allele frequencies in a population remain constant from generation to generation in the absence of disturbance including mutation, natural selection, non-random mating, genetic drift, and gene flow [11]. The detailed prediction method was described in a previous study [12]. MedCalc ver. 11.5.1.0 (MedCalc Software, Maiakerke, Belgium) was used for statistical analysis, and 95% confidence intervals (CIs) were calculated for each value.

## Results

In total, 9,197 East Asian exomes including 1,909 Korean exomes from gnomAD and 1,722 Korean genomes from KRGDB were analyzed for *RPE65* gene variants. The classification of these variants according to the 2015 ACMG/AMP guidelines, HGMD, ClinVar is summarized in Table 1. A total of nine variants were classified as PV/LPV according to the 2015 ACMG/AMP guidelines. Total carrier frequencies in East Asians and Koreans from gnomAD and KRGDB were 0.10% (11/10,919) and 0.06% (2/3,631), respectively. The estimated incidence of *RPE65*-associated IRDs was 1/3,941,308 in East Asians and 1/13,184,161 in Koreans. According to the ClinVar, carrier frequency of East Asian and Korean was 0.08% and 0.03%, respectively. The estimated incidence of *RPE65*-associated IRDs was 1/5,887,633 in East Asians and 1/52,736,644 in Koreans. According to the HGMD, carrier frequencies of East Asians and Koreans were 1.24% and 0.19%, respectively. The estimated incidence of *RPE65*-associated IRDs was 1/26,167 in East Asians and 1/1,076,258 in Koreans.

**Table 1:** Carrier frequency and estimated incidence of *RPE65* gene in East Asians and Koreans in gnomAD and KRGDB

	Variants (n)	Total individuals (n)	Carrier frequency (%), (95% CI)	Estimated incidence (1/n), (95% CI)
All East Asians (n = 10,919)				
2015 ACMG/AMP (PV/LPV)	9	11	0.10 (0.05–0.18)	1/3,941,308 (1/1,230,463–1/15,809,714)
ClinVar (PV/LPV)	7	9	0.08 (0.04–0.16)	1/5,887,633 (1/1,633,796–1/28,158,383)
HGMD (DM)	13	135	1.24 (1.04–1.46)	1/26,167 (1/18,688–1/37,197)
gnomAD East Asian exomes (n = 9,197)				
2015 ACMG/AMP (PV/LPV)	8	10	0.11 (0.05–0.20)	1/3,383,392 (1/1,000,000–1/14,736,167)
ClinVar (PV/LPV)	6	8	0.09 (0.04–0.17)	1/5,286,551 (1/1,361,724–1/28,368,744)
HGMD (DM)	12	131	1.42 (1.19–1.69)	1/19,716 (1/14,005 − 1/28,199)
All Koreans (n = 3,631)				
2015 ACMG/AMP (PV/LPV)	2	2	0.06 (0.01–0.20)	1/13,184,161 (1/1,010,380–1/899,100,675)
ClinVar (PV/LPV)	1	1	0.03 (0–0.15)	1/52,736,644 (1/1,698,737–1/81,632,653,061)
HGMD (DM)	3	7	0.19 (0.08–0.40)	1/1,076,258 (1/253,537–1/6,659,729)
gnomAD Korean exomes (n = 1,909)				
2015 ACMG/AMP (PV/LPV)	1	1	0.05 (0–0.29)	1/14,577,124 (1/469,581–1/22,612,923,286)
ClinVar (PV/LPV)	0	0	0 (0–0.19)	NA (1/1,108,033 - NA)
HGMD (DM)	2	3	0.16 (0.03–0.46)	1/1,619,680 (1/189,613–1/38,103,948)
KRGDB (n = 1,722)				
2015 ACMG/AMP (PV/LPV)	1	1	0.06 (0–0.32)	1/11,861,136 (1/382,077 − 1/18,510,805,683)
ClinVar (PV/LPV)	1	1	0.06 (0–0.32)	1/11,861,136 (1/382,077 − 1/18,510,805,683)
HGMD (DM)	2	4	0.23 (0.06–0.59)	1/741,321 (1/113,100–1/9,982,805)

*2015 ACMG/AMP* 2015 American College of Medical Genetics and Genomics and the Association for Molecular Pathology guidelines, *95% CI* 95% confidence intervals, *DM* disease-causing variant, *KRGDB* Korean Reference Genome Database, *gnomAD* Genome Aggregation Database, *LPV* likely pathogenic variant, *NA* not available, *PV* pathogenic variant

*RPE65* PVs/LPVs found in East Asians and Koreans are summarized in Table 2. Of the nine variants classified as PV/LPV identified in East Asians, only two variants were identified in Koreans: c.858 + 1G > T and c.335G > A (p.Cys112Tyr). When comparing the PVs/LPVs found in East Asians and Koreans with other ethnicities, PVs/LPVs identified in East Asians and Koreans were not found in Ashkenazi Jewish, European (Finnish), and other populations.

**Table 2:** Pathogenic variants and likely pathogenic variants of East Asians and Koreans in gnomAD and KRGDB

Nucleotide change	Amino acid change	KRGDB allele frequency (n = 1,722)	gnomAD									REVEL	2015 ACMG/AMP (criteria)	Reference
			**Korean**	**East Asian**	**African**	**Latino**	**Ashkenazi Jewish**	**European (Finnish)**	**European (non-Finnish)**	**Other**	**South Asian**			
			**(n = 1,909)**	**(n = 9,197)**	**(n = 8,128)**	**(n = 17,296)**	**(n = 5,040)**	**(n = 10,824)**	**(n = 56,885)**	**(n = 3,070)**	**(n = 15,308)**			
c.1451G > A	p.Gly484Asp	0	0	5.45E-05	0	0	0	0	9.01E-06	0	0	0.984	LPV (PM1, PM2, PM3, PP3)	[30]
c.1399 C > G	p.Pro467Ala	0	0	1.09E-04	0	0	0	0	0	0	0	0.8909	LPV (PM1, PM2, PM3, PP3)	[31]
c.858 + 1del	p.?	0	0	5.44E-05	0	0	0	0	0	0	0	-	LPV (PVS1, PM2)	[31]
c.858 + 1G > T	p.?	4.55E-04	0	0	0	0	0	0	0	0	0	-	LPV (PVS1, PM2)	[20]
c.545 A > G	p.His182Arg	0	0	5.44E-05	0	0	0	0	0	0	0	0.8999	LPV (PM1, PM2, PM3_supporting, PP3)	[32]
c.335G > A	p.Cys112Tyr	0	2.62E-04	5.44E-05	0	0	0	0	0	0	0	0.9169	LPV (PM1, PM2, PS3_moderate, PM3_supporting, PP3)	[19]
c.272G > A	p.Arg91Gln	0	0	5.44E-05	3.70E-04	0	0	0	1.76E-05	0	6.54E-05	0.6589	LPV (PM1, PM3, PM5)	[33]
c.271 C > T	p.Arg91Trp	0	0	1.09E-04	3.70E-04	2.89E-05	0	0	3.52E-05	0	3.27E-05	0.8519	LPV (PM1, PM3, PS1_moderate, PP3)	[34]
c.200T > G	p.Leu67Arg	0	0	5.44E-05	0	0	0	0	0	0	0	0.98	LPV (PM1, PM2, PM3, PP3)	[35]

*gnomAD* Genome Aggregation Database, *KRGDB* Korean Reference Genome Database, 2015 *ACMG/AMP* 2015 American College of Medical Genetics and Genomics and Association for Molecular Pathology guidelines, *LPV* likely pathogenic variant

## Discussion

In this study, the carrier frequency and estimated incidence of *RPE65*-associated IRDs were analyzed for East Asians and Koreans using gnomAD and KRGDB. The carrier frequency of East Asians was 0.10% and of Koreans was 0.06%. Based on disease classification databases, HGMD and ClinVar, carrier frequencies were 0.08–1.24% in East Asians and 0.03–0.19% in Koreans.

According to the previous study of Hanany et al., carrier frequency of IRDs in East Asians was about 40%, the second highest after Europeans, and the estimated genetic prevalence of IRDs was the highest in East Asians at 1:1,003 [13]. *EYS* and *USH2A* were the most prevalent genes in East Asians with carrier frequencies of 2.52% and 2.48%, respectively. On the other hand, *RPE65* is rare among East Asians with a carrier frequency of 0.20% [13]. The carrier frequency of *RPE65* was different in each continental population, which was highest in Africans (0.55%), followed by non-Finnish Europeans (0.28%), Latinos (0.27%), South Asians (0.20%) and Finnish (0.12%) [13]. In the present study, carrier frequency in East Asians was even lower at 0.10%. In addition, there might be difference in sub-continental population. In Koreans, the carrier frequency of *RPE65* was 0.05% in gnomAD Korean and 0.06% in KRGDB, which were lower than those of East Asians.

The carrier frequency was different according to the variant interpretation methods. The carrier frequency of 0.20% in the previous study by Hanany et al. was based on variant classification that used parameters including allele frequency, segregation analysis, biochemical analyses, presence of the variant in patients vs. controls, and biallelically vs. monoallelically [13]. In the present study, the carrier frequency based on HGMD was highest, and the carrier frequency based on 2015 ACMG/AMP guidelines and ClinVar was similar. According to the previous study of Park et al., the variants classified as DM in HGMD showed a 91.62% concordance rate compared to PV/LPV by 2015 ACMG/AMP guidelines [14]. The accuracy of variant classification in ClinVar gradually improved gradually and has been influenced by review status, represented as the number of stars [15]. Because the higher number of stars indicates a higher the concordance rate, the reliability of the classification in ClinVar can be evaluated as the number of stars. To prevent overestimation of carrier frequency, variant classification in HGMD and ClinVar should be accepted with caution. Although the 2015 ACMG/AMP guidelines were not perfect, it is considered as the standard guideline. Therefore, the carrier frequency in the present study was considered accurate to estimate the incidence of *RPE65*-associated IRDs.

According to data from the Korean Statistical Information Service (http://kosis.kr/; accessed on 10 June 2022) in 2020, the total population of Korea was 51.8 million with 272,337 births. Based on the Korean carrier frequency identified in this study, the number of carriers is estimated to be about 31,000 in total and 163 in newborns per year. The estimated incidence of *RPE65*-associated IRDs in Korea based on the Hardy-Weinberg equilibrium is approximately 0.02 cases per year. Therefore, the number of patients eligible for VN was expected to be very low in Korea.

According to the Leiden open variation database (LOVD), the most common PVs/LPVs in *RPE65* were c.271 C > T, c.1102T > C, and c.11 + 5G > A, which accounts for 26.7% of PVs/LPVs identified in *RPE65* [16]. Among them, only c.271 C > T was present in the East Asian populations. c.1102T > C is known as founder mutation in the Dutch populations but is absent in the East Asian populations [16, 17]. c.271 C > T was the most common PVs/LPVs in gnomAD East Asian, which was consistent with the findings of LOVD, but it was absent in gnomAD Korean and KRGDB. c.271 C > T was frequently identified in Saudi Arabia and Tunisia [16] and was identified in one Korean patient with LCA [18]. Only one PV/LPV was present in gnomAD Korean and in KRGDB: c.335G > A in gnomAD Korean and c.858 + 1G > T in KRGDB. c.335G > A was identified as homozygous in a Chinese patient with autosomal recessive RP [19]. c.858 + 1G > T was identified as homozygous in two Indian families with LCA [20, 21] and in one Korean LCA patient as compound heterozygous with c.271 C > T [18]. Based on these findings, it was found that the variant spectrum of *RPE65* varies in different ethnicities, and Koreans showed a variant spectrum similar to that of the overall Asian populations.

Genotype-phenotype correlation analysis identified that truncating variants were associated with severe phenotype and early disease onset [22, 23]. Most of the PVs/LPVs identified in the East Asian database consisted of missense variant, and splicing variants accounted for only a small portion. In addition, the most common variants were missense variants: c.271 C > T and c.1399 C > G. Therefore, the variant spectrum in the patients of *RPE65*-associated IRDs was expected to consist mostly of missense variants. This was consistent with LOVD, in which the most common variant is the missense variant, c.271 C > T. Indeed, previous studies showed that missense variants were most frequent in patients of *RPE65*-associated IRDs regardless of ethnicity [22, 23].

Since a treatment has been approved for *RPE65*-associated IRDs, identification of *RPE65* patients has become important. Recently, the *RPE65* gene was added in ACMG secondary findings v3.0 because gene therapy treatment that may be more efficacious earlier in disease progression of patients is helpful in early diagnosis [24]. Therefore, it is meaningful to predict the carrier and prevalence of *RPE65*-associated IRDs in East Asians and Koreans through this study.

This study has some limitations. First, we did not analyze structural variations such as large deletion/insertion of the *RPE65* gene. Currently, only two cases of large deletion of *RPE65* have been reported [25, 26]. Second, we analyzed two separate population databases, gnomAD and KRGDB, of which the strategies for variants calling and filtering can differ. It might affect the carrier frequency calculated using each database. Third, although rare, *RPE65* was associated with autosomal dominant RP 87 with choroidal involvement (RP87), which was characterized with incomplete penetrance and variable expressivity [27, 28]. Therefore, the carrier frequency estimated in this study might include patients with RP87 as well as carriers of *RPE65*-associated IRD. To date, only one causative variant, c.1430 A > G, was identified to cause RP87 [27, 29], which was not identified in this study. Nonetheless, this study has several advantages. This is the largest study among those performed in East Asia that analyzed the entire *REP65* gene. To the best of our knowledge, there are no large-scale population studies of carrier frequency and estimated *RPE65*-associated IRDs incidence in Koreans. We believe that this study more accurately predicts the carrier frequency of *RPE65*-associated IRDs in East Asia and Korea.

## Conclusion

This study identified the carrier frequencies in East Asians and Koreans using gnomAD and KRGDB. We confirmed that the carrier frequency of *RPE65*-associated IRDs patients was low in Koreans among East Asians, and the incidence was also predicted to be lower than in other East Asian populations. The variant spectrum of *RPE65* genes in East Asian and Korean populations differed greatly from those of other ethnic groups. Our data are expected to serve as a reference for further investigations of *RPE65*-associated IRDs in East Asian and Korean populations.

## Data Availability

All data are available by corresponding author upon reasonable request.

## Abbreviations

2015 ACMG/AMP guidelines: 2015 American College of Medical Genetics and Genomics and the Association for Molecular Pathology guideline
ACMG: American College of Medical Genetics
CIs: Confidence intervals
FDA: Food and Drug Administration
DM: Disease-causing variant
gnomAD: Genome Aggregation Database
HGMD: Human Gene Mutation Database
IRD: Inherited retinal diseases
KRGDB: Korean Reference Genome Database
LCA: Leber congenital amaurosis
LOVD: Leiden open variation database
LPV: Likely pathogenic variant
PV: Pathogenic variant
PR: Retinitis pigmentosa
VN: Voretigene neparvovec-rzyl

## References

[1] Berger W, Kloeckener-Gruissem B, Neidhardt J (2010). The molecular basis of human retinal and vitreoretinal diseases. Prog Retin Eye Res.

[2] RetNet. Summaries of genes and loci causing retinal diseases. https://sph.uth.edu/retnet/.

[3] Redmond TM, Yu S, Lee E, Bok D, Hamasaki D, Chen N, Goletz P, Ma JX, Crouch RK, Pfeifer K (1998). Rpe65 is necessary for production of 11-cis-vitamin A in the retinal visual cycle. Nat Genet.

[4] Sallum JMF, Kaur VP, Shaikh J, Banhazi J, Spera C, Aouadj C, Viriato D, Fischer MD (2022). Epidemiology of Mutations in the 65-kDa Retinal Pigment Epithelium (RPE65) Gene-Mediated Inherited Retinal Dystrophies: A Systematic Literature Review. Adv Ther.

[5] LUXTURNATM (voretigene neparvovec-rzyl) December 19, 2017 approval letter. 2017. https://www.fda.gov/downloads/BiologicsBloodVaccines/CellularGeneTherapyProducts/ApprovedProducts/UCM589690.pdf

[6] Karczewski KJ, Francioli LC, Tiao G, Cummings BB, Alföldi J, Wang Q, Collins RL, Laricchia KM, Ganna A, Birnbaum DP (2020). The mutational constraint spectrum quantified from variation in 141,456 humans. Nature.

[7] Jung KS, Hong KW, Jo HY, Choi J, Ban HJ, Cho SB, Chung M: KRGDB: the large-scale variant database of 1722 Koreans based on whole genome sequencing. *Database (Oxford)* 2020, 2020. 10.1093/database/baaa030 PMC7190023 32348452

[8] Richards S, Aziz N, Bale S, Bick D, Das S, Gastier-Foster J, Grody WW, Hegde M, Lyon E, Spector E (2015). Standards and guidelines for the interpretation of sequence variants: a joint consensus recommendation of the American College of Medical Genetics and Genomics and the Association for Molecular Pathology. Genet Med.

[9] Ioannidis NM, Rothstein JH, Pejaver V, Middha S, McDonnell SK, Baheti S, Musolf A, Li Q, Holzinger E, Karyadi D (2016). REVEL: An Ensemble Method for Predicting the Pathogenicity of Rare Missense Variants. Am J Hum Genet.

[10] Jaganathan K, Kyriazopoulou Panagiotopoulou S, McRae JF, Darbandi SF, Knowles D, Li YI, Kosmicki JA, Arbelaez J, Cui W, Schwartz GB (2019). Predicting Splicing from Primary Sequence with Deep Learning. Cell.

[11] Edwards AW (2008). G. H. Hardy (1908) and Hardy-Weinberg equilibrium. Genetics.

[12] Park JE, Lee T, Ha K, Ki CS (2021). Carrier frequency and incidence estimation of Smith-Lemli-Opitz syndrome in East Asian populations by Genome Aggregation Database (gnomAD) based analysis. Orphanet J Rare Dis.

[13] Hanany M, Rivolta C, Sharon D (2020). Worldwide carrier frequency and genetic prevalence of autosomal recessive inherited retinal diseases. Proc Natl Acad Sci U S A.

[14] Park KJ, Lee W, Chun S, Min WK (2021). The Frequency of Discordant Variant Classification in the Human Gene Mutation Database: A Comparison of the American College of Medical Genetics and Genomics Guidelines and ClinVar. Lab Med.

[15] Rehm HL, Harrison SM, Martin CL (2017). ClinVar Is a Critical Resource to Advance Variant Interpretation. Oncologist.

[16] Astuti GD, Bertelsen M, Preising MN, Ajmal M, Lorenz B, Faradz SM, Qamar R, Collin RW, Rosenberg T, Cremers FP (2016). Comprehensive genotyping reveals RPE65 as the most frequently mutated gene in Leber congenital amaurosis in Denmark. Eur J Hum Genet.

[17] Yzer S, van den Born LI, Schuil J, Kroes HY, van Genderen MM, Boonstra FN, van den Helm B, Brunner HG, Koenekoop RK, Cremers FP (2003). A Tyr368His RPE65 founder mutation is associated with variable expression and progression of early onset retinal dystrophy in 10 families of a genetically isolated population. J Med Genet.

[18] Seong MW, Kim SY, Yu YS, Hwang JM, Kim JY, Park SS (2008). Molecular characterization of Leber congenital amaurosis in Koreans. Mol Vis.

[19] Liu X, Tao T, Zhao L, Li G, Yang L (2021). Molecular diagnosis based on comprehensive genetic testing in 800 Chinese families with non-syndromic inherited retinal dystrophies. Clin Exp Ophthalmol.

[20] Gu SM, Thompson DA, Srikumari CR, Lorenz B, Finckh U, Nicoletti A, Murthy KR, Rathmann M, Kumaramanickavel G, Denton MJ (1997). Mutations in RPE65 cause autosomal recessive childhood-onset severe retinal dystrophy. Nat Genet.

[21] Srilekha S, Arokiasamy T, Srikrupa NN, Umashankar V, Meenakshi S, Sen P, Kapur S, Soumittra N (2015). Homozygosity Mapping in Leber Congenital Amaurosis and Autosomal Recessive Retinitis Pigmentosa in South Indian Families. PLoS ONE.

[22] Lopez-Rodriguez R, Lantero E, Blanco-Kelly F, Avila-Fernandez A, Martin Merida I, Del Pozo-Valero M, Perea-Romero I, Zurita O, Jiménez-Rolando B, Swafiri ST (2021). RPE65-related retinal dystrophy: Mutational and phenotypic spectrum in 45 affected patients. Exp Eye Res.

[23] Gao FJ, Wang DD, Li JK, Hu FY, Xu P, Chen F, Qi YH, Liu W, Li W, Zhang SH (2021). Frequency and phenotypic characteristics of RPE65 mutations in the Chinese population. Orphanet J Rare Dis.

[24] Miller DT, Lee K, Chung WK, Gordon AS, Herman GE, Klein TE, Stewart DR, Amendola LM, Adelman K, Bale SJ (2021). ACMG SF v3.0 list for reporting of secondary findings in clinical exome and genome sequencing: a policy statement of the American College of Medical Genetics and Genomics (ACMG). Genet Med.

[25] Ellingford JM, Horn B, Campbell C, Arno G, Barton S, Tate C, Bhaskar S, Sergouniotis PI, Taylor RL, Carss KJ (2018). Assessment of the incorporation of CNV surveillance into gene panel next-generation sequencing testing for inherited retinal diseases. J Med Genet.

[26] Al-Gazali L, Ali BR (2010). Mutations of a country: a mutation review of single gene disorders in the United Arab Emirates (UAE). Hum Mutat.

[27] Hull S, Mukherjee R, Holder GE, Moore AT, Webster AR (2016). The clinical features of retinal disease due to a dominant mutation in RPE65. Mol Vis.

[28] Li Y, Furhang R, Ray A, Duncan T, Soucy J, Mahdi R, Chaitankar V, Gieser L, Poliakov E, Qian H (2019). Aberrant RNA splicing is the major pathogenic effect in a knock-in mouse model of the dominantly inherited c.1430A > G human RPE65 mutation. Hum Mutat.

[29] Jauregui R, Park KS, Tsang SH (2018). Two-year progression analysis of RPE65 autosomal dominant retinitis pigmentosa. Ophthalmic Genet.

[30] Weleber RG, Michaelides M, Trzupek KM, Stover NB, Stone EM (2011). The phenotype of Severe Early Childhood Onset Retinal Dystrophy (SECORD) from mutation of RPE65 and differentiation from Leber congenital amaurosis. Invest Ophthalmol Vis Sci.

[31] Zhong Z, Rong F, Dai Y, Yibulayin A, Zeng L, Liao J, Wang L, Huang Z, Zhou Z, Chen J (2019). Seven novel variants expand the spectrum of RPE65-related Leber congenital amaurosis in the Chinese population. Mol Vis.

[32] Jacobson SG, Aleman TS, Cideciyan AV, Sumaroka A, Schwartz SB, Windsor EA, Traboulsi EI, Heon E, Pittler SJ, Milam AH (2005). Identifying photoreceptors in blind eyes caused by RPE65 mutations: Prerequisite for human gene therapy success. Proc Natl Acad Sci U S A.

[33] Thompson DA, Gyürüs P, Fleischer LL, Bingham EL, McHenry CL, Apfelstedt-Sylla E, Zrenner E, Lorenz B, Richards JE, Jacobson SG (2000). Genetics and phenotypes of RPE65 mutations in inherited retinal degeneration. Invest Ophthalmol Vis Sci.

[34] Morimura H, Fishman GA, Grover SA, Fulton AB, Berson EL, Dryja TP (1998). Mutations in the RPE65 gene in patients with autosomal recessive retinitis pigmentosa or leber congenital amaurosis. Proc Natl Acad Sci U S A.

[35] Xu F, Dong Q, Liu L, Li H, Liang X, Jiang R, Sui R, Dong F (2012). Novel RPE65 mutations associated with Leber congenital amaurosis in Chinese patients. Mol Vis.

